# Moderate-to-Vigorous Physical Activity and School Engagement in Adolescents Attending Rural and Urban Schools in Southern Spain

**DOI:** 10.3390/children13081074

**Published:** 2026-08-13

**Authors:** José Luis Solas-Martínez, Pablo Ramírez-Espejo, Rosa Gómez-Martínez, Alberto Ruiz-Ariza

**Affiliations:** Department of Didactic of Musical, Plastic, and Corporal Expression, University of Jaén, 23071 Jaén, Spain; jsolas@ujaen.es (J.L.S.-M.); rgm00094@red.ujaen.es (R.G.-M.); arariza@ujaen.es (A.R.-A.)

**Keywords:** moderate-to-vigorous physical activity, school engagement, cognitive engagement, adolescents, rural schools, urban schools

## Abstract

**Highlights:**

**What are the main findings?**

- Active adolescents from an urban context showed higher cognitive engagement than their inactive peers.
- Lower weekly MVPA was associated with higher odds of low cognitive engagement in the urban subgroup.

**What is the implication of the main finding?**

- Promoting regular moderate-to-vigorous physical activity may be relevant for supporting cognitive aspects of school engagement during adolescence.
- Further research is needed to determine whether rural–urban context modifies the relationship between MVPA and school-related outcomes.

**Abstract:**

**Background/Objectives:** Adolescence is a key period for the development of physical activity habits and school-related behaviors. Moderate-to-vigorous physical activity (MVPA) has been associated with cognitive and educational outcomes, but its relationship with different dimensions of school engagement in rural and urban contexts remains unclear. This study aimed to examine the association between weekly MVPA and behavioral, emotional, and cognitive engagement among adolescents attending rural and urban schools in southern Spain. **Methods:** A cross-sectional quantitative study was conducted with 282 adolescents (199 from urban schools and 83 from rural schools). School engagement was assessed using the Spanish version of the School Engagement Measure, and weekly MVPA was measured using the PACE+ Adolescent Physical Activity Measure. Adolescents were classified as active or inactive according to whether they reported at least five days per week of MVPA. ANCOVA and binary logistic regression analyses were performed separately by school context, adjusting for age, sex, and BMI. Complementary interaction analyses were conducted to test whether the associations differed across school contexts. **Results:** No significant differences were found between rural and urban adolescents in MVPA or school engagement. In urban schools, active adolescents showed significantly higher cognitive engagement than inactive adolescents. Lower weekly MVPA was also associated with higher odds of low cognitive engagement in urban adolescents. No significant associations were observed for behavioral or emotional engagement, or among rural adolescents. **Conclusions:** In subgroup-specific analyses, weekly MVPA was associated with cognitive engagement among adolescents attending urban schools. Given the cross-sectional design, convenience sampling, and self-reported assessment of MVPA, these findings should be interpreted as correlational and cannot establish causality. Moreover, the interaction analyses provided no evidence that the association differed significantly between rural and urban school contexts.

## 1. Introduction

Adolescence is a key developmental period for the consolidation of health-related behaviors, including regular physical activity. Moderate-to-vigorous physical activity (MVPA) is particularly relevant because it represents the intensity range most strongly associated with physical, psychological, and developmental benefits in young people [1,2]. International guidelines recommend that children and adolescents accumulate an average of at least 60 min per day of MVPA across the week [3]. However, insufficient physical activity remains highly prevalent among adolescents worldwide, with global surveillance data showing that most school-going adolescents do not meet recommended activity levels [2,4,5]. In Spain, recent regional evidence has also reported low compliance with international physical activity and sedentary behavior recommendations among children and adolescents [6]. Moreover, recent evidence from southern Spain has shown that sedentary behavior patterns may be associated with school engagement among young people [7]. These habits may vary according to the context in which adolescents live, since physical activity, outdoor play, and screen-time trends can differ across rural and urban settings [8,9,10]. In rural Spanish adolescents, family and friend support has been shown to be relevant for explaining physical activity levels, suggesting that social support may play an important role in active behaviors in these contexts [11]. Therefore, rural–urban differences in activity habits, sedentary behaviors, and social support may be important not only for understanding adolescents’ health behaviors, but also for examining how these behaviors relate to educational outcomes.

Beyond its relevance for physical health, physical activity has been increasingly studied in relation to educational and cognitive outcomes [12,13,14]. MVPA is particularly relevant within this field because it represents the intensity range emphasized in international physical activity recommendations for children and adolescents and produces a greater cardiorespiratory and metabolic stimulus than lower-intensity activity [1,2,3]. This stimulus may promote acute neurobiological responses, including increased cerebral blood flow, prefrontal cortex activation, and neurotrophic signaling involving brain-derived neurotrophic factor, which may support learning-related cognitive processes [15,16]. Recent evidence suggests that physical activity interventions may improve executive functions in children and adolescents, particularly inhibitory control and working memory. These functions are closely related to students’ capacity to sustain attention, resist distractions, organize behavior, and persist in learning tasks [17,18,19]. Accordingly, the neurobiological and executive responses associated with MVPA may facilitate the attention, effort, and strategic involvement that characterize cognitive engagement [20,21]. MVPA may be associated with cognitive engagement through psychological mechanisms such as self-regulation and self-efficacy, which help students manage learning demands, sustain effort, persist, and engage strategically in learning [22]. In addition, physical exercise may support perceived competence and coping with academic demands through psychological resilience, stress self-management, and mental health-related resources [23,24]. Taken together, these mechanisms suggest a stepwise pathway whereby MVPA may support executive and psychological resources that subsequently facilitate cognitive engagement [15,22,25].

Within the educational field, school engagement is an important construct for understanding students’ involvement in school and learning. School engagement is commonly conceptualized as a multidimensional construct composed of behavioral, emotional, and cognitive dimensions [20,26,27]. Behavioral engagement refers to participation, attention, effortful behavior, and adherence to school norms [28,29]. Emotional engagement reflects students’ affective connection with school, including belonging, interest, enjoyment, and perceived connection with teachers and peers [30]. Cognitive engagement refers to students’ psychological investment in learning, persistence, strategic involvement, self-regulation, and willingness to make the effort required to understand complex ideas [27,28]. Recent research has reinforced the importance of identifying factors that may promote higher levels of school engagement during adolescence, given its relevance for learning, achievement, and positive development [28]. Moreover, recent evidence suggests that behavioral, emotional, and cognitive engagement, although interrelated, do not necessarily evolve in parallel and may differ in their subject specificity, temporal stability, and educational functions [27,30]. Therefore, examining how MVPA is associated with these dimensions may help clarify its potential contribution to school engagement during adolescence.

Previous systematic review and meta-analytic evidence suggest a small positive association between physical activity and school engagement in young people, although findings appear to be heterogeneous [25]. This heterogeneity may be partly explained by differences in how physical activity is assessed, the engagement dimensions considered, and the contextual characteristics of the samples studied [25,28]. From a multidimensional perspective, MVPA could be related to the three dimensions of school engagement through different pathways. Behavioral engagement may be influenced by the structured, participatory, and rule-based nature of many physical activity and sport contexts, which can promote involvement, effort, and participation in school-related routines. Emotional engagement may also be supported by the social and affective experiences associated with physical activity, including peer interaction, perceived support, enjoyment, and feelings of belonging [31,32]. Cognitive engagement may be linked to the self-regulatory and motivational processes involved in regular physical activity, such as persistence, self-efficacy, perceived competence, and strategic effort in learning situations [17,22,23]. Therefore, analyzing behavioral, emotional, and cognitive engagement separately may help clarify whether MVPA is similarly associated with all dimensions of school engagement or whether its relationship differs according to the specific dimension considered.

The rural–urban context may also be relevant when examining the association between MVPA and school engagement, as adolescents’ movement behaviors, screen exposure, access to green spaces, and social support may differ according to urbanicity [9,11,33]. Rural and urban environments may also differ in access to sport facilities and organized activities, transport opportunities, perceived safety, neighborhood amenities, and family or peer support, all of which have been associated with physical activity participation among young people [8,11,33,34,35]. These contextual characteristics may influence not only opportunities to engage in MVPA but also the conditions and psychosocial experiences accompanying it [11,22,34,35]. For instance, differences in social support, structured sport participation, or the extent to which MVPA replaces screen-based or other sedentary behaviors may affect the self-regulatory, motivational, and interpersonal processes through which physical activity relates to educational outcomes [8,9,22]. Consequently, similar levels of MVPA may occur within different social and environmental contexts and may therefore show different associations with school engagement in rural and urban adolescents [22,25,34]. In addition, individual characteristics such as age, sex, and BMI should be considered when examining this association. Age is relevant because adolescence involves developmental, motivational, and academic transitions [28], sex because girls generally report higher levels of insufficient physical activity than boys [5], and BMI because it is related to physical activity, health status, and school-related outcomes such as academic achievement and classroom participation [36].

Despite growing evidence linking physical activity with cognitive and educational outcomes, much of the literature has focused on academic achievement, cognitive function, or psychological mechanisms rather than specifically on school engagement [13,18,37]. Moreover, school engagement is not always analyzed through its behavioral, emotional, and cognitive dimensions, despite evidence showing that these dimensions may differ in their stability, antecedents, and educational functions [21,27,30]. Limited evidence has also examined whether the association between MVPA and school engagement differs between rural and urban adolescents, even though movement behaviors, screen-time patterns, environmental opportunities, and social support may vary according to context. Therefore, the aim of this study was to examine the association between weekly MVPA and school engagement among adolescents from rural and urban schools in southern Spain. Specifically, this study analyzed whether active and inactive adolescents differed in behavioral, emotional, and cognitive engagement according to school context, and whether lower weekly MVPA was associated with higher odds of low school engagement in rural and urban adolescents. Accordingly, the primary hypothesis was that higher weekly MVPA would be associated with higher levels of school engagement across its behavioral, emotional, and cognitive dimensions. The secondary hypothesis was that the magnitude of these associations would differ according to rural or urban school context.

## 2. Materials and Methods

### 2.1. Design and Participants

This cross-sectional quantitative study was conducted with adolescents enrolled in six educational institutions in southern Spain. Three educational centers were located in urban municipalities and three in rural municipalities. Schools were selected through convenience sampling according to their availability and interest in participating in the research project. School context was classified using the 10,000-inhabitant threshold because the municipality was the predefined unit of classification, and this criterion has been used in Spanish demographic research [38]. The final sample included 282 adolescents, of whom 199 attended urban schools and 83 attended rural schools. No a priori statistical power analysis was conducted because the final sample size was determined by the availability of the participating schools and eligible students.

### 2.2. Measures

#### 2.2.1. Dependent Variables: School Engagement

School engagement was assessed using the Spanish version of the School Engagement Measure (SEM), originally developed by Fredricks et al. [39] and validated in Spanish adolescents by Ramos-Díaz et al. [40]. This instrument evaluates school engagement as a multidimensional construct composed of three dimensions: behavioral engagement, emotional engagement, and cognitive engagement.

The SEM consists of 19 items answered on a 5-point Likert-type scale ranging from 1 = never to 5 = always. The behavioral engagement dimension includes five items related to students’ participation, attention, and appropriate behavior in the school context. The emotional engagement dimension includes six items assessing students’ affective connection with school, including feelings of belonging, interest, and enjoyment. The cognitive engagement dimension includes eight items evaluating students’ psychological investment in learning, effort, and use of learning strategies.

For the present study, mean scores were calculated separately for behavioral, emotional, and cognitive engagement. Higher scores indicated higher levels of engagement in each dimension. The Spanish validation of the SEM supported a three-dimensional structure composed of behavioral, emotional, and cognitive engagement, with acceptable fit indices (CFI = 0.906; RMSEA = 0.068; SRMR = 0.058). Internal consistency values were adequate, with Cronbach’s alpha coefficients of α = 0.74 for behavioral engagement, α = 0.81 for emotional engagement, and α = 0.77 for cognitive engagement [40]. In the current sample, Cronbach’s alpha coefficients were α = 0.70 for behavioral engagement, α = 0.87 for emotional engagement, and α = 0.78 for cognitive engagement.

#### 2.2.2. Predictor/Independent Variables: Weekly Moderate-to-Vigorous Physical Activity

MVPA was considered the main predictor variable. This variable was assessed using the PACE+ Adolescent Physical Activity Measure [41], which consists of two items asking participants to report the number of days on which they engaged in at least 60 min of MVPA during the previous week and during a typical week. The final MVPA score was calculated as the average of both items: (P1 + P2)/2, with higher scores indicating a greater number of weekly MVPA days. The internal consistency of this measure was acceptable (α = 0.79) [41]. Weekly MVPA was also dichotomized into two groups: active adolescents, defined as those reporting ≥5 days/week of MVPA, and inactive adolescents, defined as those reporting <5 days/week of MVPA.

#### 2.2.3. Covariates

Age, sex, and BMI were included as covariates in the adjusted analyses because they may influence the main variables examined in this study. Participants’ age and sex were collected using a sociodemographic questionnaire completed by the students. Age was reported in years and was included as a covariate because school engagement may vary across adolescence, showing developmental changes in behavioral, emotional, and cognitive engagement during secondary school [42]. Sex was recorded as a categorical variable and included as a covariate because physical activity patterns differ between boys and girls during adolescence, with global evidence showing higher levels of insufficient physical activity among girls than boys [5].

BMI was included as a covariate due to its relationship with physical activity, health status, and school-related outcomes. Previous research has shown that higher BMI is associated with lower academic achievement and lower classroom participation, supporting its relevance in studies examining school engagement [36]. Body mass index was calculated using Quetelet’s formula: weight (kg)/height^2^ (m^2^). Body weight was measured using a digital ASIMED^®^ Type B, Class III scale, and height was measured using a SECA^®^ 214 portable stadiometer (SECA Ltd., Hamburg, Germany). All anthropometric measurements were taken with participants wearing light clothing and no footwear to improve measurement accuracy. For descriptive purposes, BMI-for-age categories were additionally determined according to participants’ sex and age. Participants were classified as having thinness (<−2 SD), normal weight (≥−2 to ≤+1 SD), overweight (>+1 to ≤+2 SD), or obesity (>+2 SD) [43].

### 2.3. Procedure

Before data collection, the research team informed participating schools, teachers, families, and students about the objectives and procedures of the study. Written informed consent was obtained from parents or legal guardians, and students were informed that participation was voluntary, anonymous, and confidential. Data were collected during regular school hours, according to the schedule agreed with each school. Questionnaires were completed in the students’ usual classrooms under the supervision of trained researchers and classroom tutors. Standardized instructions were provided before completion, and researchers were available to resolve procedural doubts without influencing students’ responses. Each participant was assigned an identification code to ensure anonymity, and no personal identifying information was included in the database used for analysis. No financial, academic, or other incentives were provided to schools, families, or students for participation. This study was approved by the Bioethics Committee of the University of Jaén, Spain (reference: NOV.22/2.PRY). The study was conducted in accordance with Spanish regulations on research involving human participants, including Law 14/2007, of July 3, on Biomedical Research, and current regulations on personal data protection, including Regulation (EU) 2016/679 and Organic Law 3/2018, of December 5, on Personal Data Protection and Guarantee of Digital Rights. The study also followed the ethical principles of the Declaration of Helsinki.

### 2.4. Statistical Analysis

Descriptive analyses were performed for the total sample and separately according to school context (urban and rural). Continuous variables are presented as means and standard deviations, whereas categorical variables are presented as percentages. Student’s *t*-tests were used to compare continuous variables between urban and rural adolescents, and the Chi-square (χ^2^) test was applied to compare categorical variables. BMI-for-age categories were used only for descriptive purposes, whereas BMI was retained as a continuous covariate in all adjusted analyses. The assumptions of normality and homogeneity of variances were assessed using the Kolmogorov–Smirnov test and Levene’s test, respectively. Missing data were handled using analysis-specific listwise deletion; therefore, participants with missing values in any variable required for a particular analysis were excluded only from that analysis. The proportion of excluded participants was low, ranging from 0.4% to 1.8% across the analyses conducted for the three engagement dimensions. Given the limited amount of missing data, imputation was not considered necessary.

Furthermore, to formally test whether the associations differed according to school context, complementary interaction analyses were conducted. The ANCOVA models included the physical activity status × school context interaction, whereas the logistic regression models included the mean-centered weekly MVPA × school context interaction. All interaction models were adjusted for age, BMI, and sex.

Weekly MVPA was analyzed using both categorical and continuous approaches. For the categorical analyses, weekly MVPA was dichotomized into two groups: active adolescents, defined as those reporting ≥5 days/week of MVPA, and inactive adolescents, defined as those reporting <5 days/week of MVPA. This categorization was used to examine differences in school engagement according to physical activity status. To analyze differences in behavioral, emotional, and cognitive engagement between active and inactive adolescents, analyses of covariance (ANCOVA) were conducted. Each school engagement dimension was entered as a dependent variable in separate models. Physical activity status was included as the fixed factor, and age, BMI, and sex were included as covariates. These analyses were performed separately for urban and rural adolescents to obtain context-specific adjusted estimates. Estimated marginal means and their corresponding 95% confidence intervals were reported for active and inactive adolescents. Partial eta squared (partial η^2^) was reported as the effect size for the physical activity status factor and interpreted as small (0.01), medium (0.06), or large (0.14). Given the unequal sample sizes between active and inactive groups, Hedges’ g was calculated as an additional effect size for mean differences. Effect sizes were interpreted as small (0.2), medium (0.5), and large (0.8). In addition, the percentage difference between active and inactive adolescents was calculated for significant comparisons using the following formula: [(higher mean − lower mean)/lower mean] × 100.

Binary logistic regression analyses were performed to examine the association between lower weekly MVPA and the odds of low school engagement. Weekly MVPA was retained as a continuous predictor and was not dichotomized. To define the outcome categories, each school engagement dimension was dichotomized using the sample median as the cut-off point, as no established clinical or educational thresholds are available for the SEM dimensions. This approach has been used in recent school-engagement research to operationalize lower and higher engagement groups and facilitate the interpretation of between-group comparisons [44]. Nevertheless, median dichotomization may reduce information and statistical power and introduce bias through an arbitrary, sample-dependent cut-off. Participants scoring below the median were classified as having low engagement, whereas those scoring at or above the median were classified as having high engagement and used as the reference group. Separate logistic regression models were fitted for behavioral, emotional, and cognitive engagement. To facilitate interpretation, weekly MVPA was reverse-scored by subtracting the original score from seven. Consequently, a one-unit increase in the reverse-scored variable represented one fewer day per week of MVPA. Odds ratios greater than one therefore indicated higher odds of low engagement for each one-day decrease in weekly MVPA. Age, BMI, and sex were included as covariates in all logistic regression models. Analyses were conducted separately for urban and rural adolescents. Model fit was evaluated for each logistic regression model using Nagelkerke’s R^2^ and the Hosmer–Lemeshow goodness-of-fit test. A non-significant Hosmer–Lemeshow test was interpreted as indicating no evidence of poor model calibration.

All analyses were conducted using IBM SPSS Statistics, version 25.0 for Windows (IBM Corp., Armonk, NY, USA). Statistical significance was set at *p* < 0.05, and odds ratios (OR) with 95% confidence intervals (95% CI) were reported for logistic regression analyses.

## 3. Results

### 3.1. Participant Characteristics by School Context

A total of 282 adolescents were included in the descriptive analysis, of whom 199 were from urban schools and 83 from rural schools. Descriptive characteristics of the total sample and according to school context are presented in Table 1. No statistically significant differences were observed between urban and rural adolescents in age, weight, height, BMI, MVPA, or any of the school engagement dimensions (all *p* > 0.05). Regarding BMI-for-age categories, 2.1% of participants were classified as having thinness, 64.1% as normal weight, 24.2% as overweight, and 9.6% as having obesity; the distribution of these categories did not differ significantly between urban and rural adolescents (*p* = 0.483). Likewise, the proportion of adolescents classified as active was similar between urban and rural contexts (37.7% vs. 34.9%, respectively; *p* = 0.764).

### 3.2. Interaction Between Physical Activity and School Context

Complementary interaction analyses were conducted to formally examine whether the associations between physical activity and school engagement differed according to school context. The physical activity status × school context interaction was not statistically significant for behavioral engagement, F(1, 274) = 0.164, *p* = 0.686, partial η^2^ = 0.001; emotional engagement, F(1, 271) = 0.008, *p* = 0.930, partial η^2^ < 0.001; or cognitive engagement, F(1, 270) = 0.588, *p* = 0.444, partial η^2^ = 0.002. Similarly, the weekly MVPA × school context interaction was not significant in the logistic regression models for behavioral engagement, OR = 0.859, 95% CI [0.622, 1.188], *p* = 0.358; emotional engagement, OR = 0.923, 95% CI [0.668, 1.275], *p* = 0.626; or cognitive engagement, OR = 1.012, 95% CI [0.732, 1.400], *p* = 0.941. Therefore, the magnitude of the associations did not differ significantly between urban and rural school contexts.

### 3.3. Differences in School Engagement Between Active and Inactive Adolescents According to School Context

**Figure 1:**
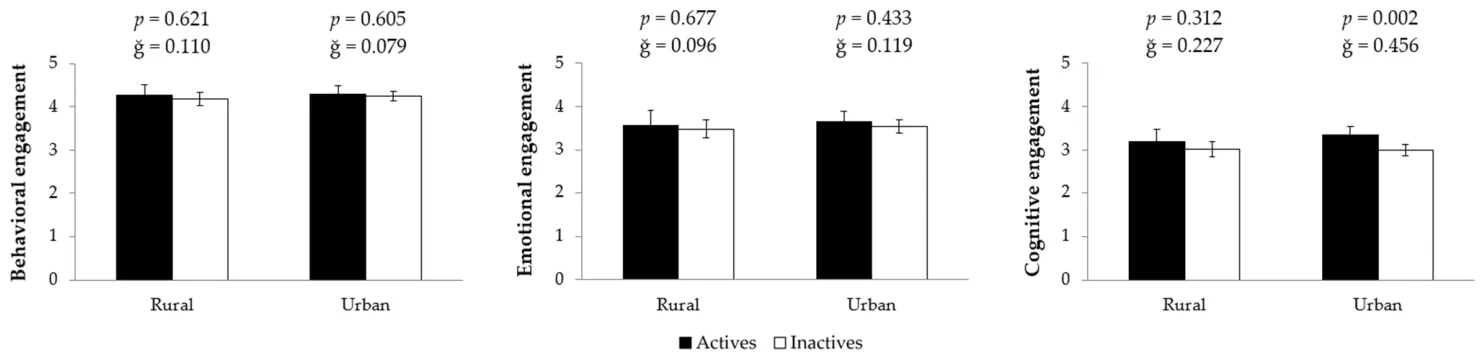
Differences in behavioral, emotional, and cognitive engagement between active and inactive adolescents according to school context. Note. Analyses were adjusted for age, BMI, and sex. Bars represent adjusted means, and error bars represent 95% confidence intervals. Hedges’ g values indicate the magnitude of differences between active and inactive adolescents within each context.

ANCOVA analyses were conducted to examine differences in behavioral, emotional, and cognitive engagement between active and inactive adolescents separately for rural and urban school contexts. As shown in Figure 1, no significant differences were observed in behavioral engagement in either rural schools (active: adjusted mean = 4.268, 95% CI [4.020, 4.516] vs. inactive: adjusted mean = 4.189, 95% CI [4.011, 4.368], *p* = 0.621, partial η^2^ = 0.003, Hedges’ g = 0.110) or urban schools (active: adjusted mean = 4.302, 95% CI [4.154, 4.449] vs. inactive: adjusted mean = 4.252, 95% CI [4.139, 4.365], *p* = 0.605, partial η^2^ = 0.001, Hedges’ g = 0.079). Similarly, no significant differences were found in emotional engagement in rural schools (active: adjusted mean = 3.575, 95% CI [3.231, 3.919] vs. inactive: adjusted mean = 3.483, 95% CI [3.233, 3.734], *p* = 0.677, partial η^2^ = 0.002, Hedges’ g = 0.096) or urban schools (active: adjusted mean = 3.651, 95% CI [3.443, 3.859] vs. inactive: adjusted mean = 3.545, 95% CI [3.386, 3.703], *p* = 0.433, partial η^2^ = 0.003, Hedges’ g = 0.119).

Regarding cognitive engagement, active adolescents showed higher adjusted mean scores than inactive adolescents in both contexts. In rural schools, active adolescents showed slightly higher cognitive engagement than inactive adolescents, although the difference was not statistically significant (active: adjusted mean = 3.206, 95% CI [2.936, 3.477] vs. inactive: adjusted mean = 3.030, 95% CI [2.831, 3.228], *p* = 0.312, partial η^2^ = 0.013, Hedges’ g = 0.227). In contrast, active adolescents in urban schools showed significantly higher cognitive engagement than their inactive peers, with an approximately 11.4% higher adjusted mean score and a small-to-moderate effect size (active: adjusted mean = 3.347, 95% CI [3.175, 3.519] vs. inactive: adjusted mean = 3.005, 95% CI [2.874, 3.136], *p* = 0.002, partial η^2^ = 0.047, Hedges’ g = 0.456).

### 3.4. Risk of Low School Engagement Associated with Lower Weekly MVPA According to School Context

Binary logistic regression analyses were conducted to examine whether lower weekly MVPA was associated with a higher risk of low school engagement, separately for rural and urban adolescents. As shown in Table 2, lower weekly MVPA was not significantly associated with low behavioral engagement in either rural adolescents (OR = 1.122, 95% CI = 0.806–1.565, *p* = 0.494) or urban adolescents (OR = 1.135, 95% CI = 0.951–1.355, *p* = 0.163). Similarly, no statistically significant associations were observed for low emotional engagement in rural adolescents (OR = 1.245, 95% CI = 0.853–1.818, *p* = 0.256) or urban adolescents (OR = 1.211, 95% CI = 0.981–1.493, *p* = 0.075), although the latter showed a tendency toward significance.

Regarding cognitive engagement, lower weekly MVPA was not significantly associated with low cognitive engagement among rural adolescents (OR = 1.355, 95% CI = 0.898–2.041, *p* = 0.148). However, among urban adolescents, each one-day decrease in weekly MVPA was significantly associated with higher odds of low cognitive engagement (OR = 1.300, 95% CI = 1.067–1.585, *p* = 0.009). This indicates that, in urban adolescents, lower weekly MVPA was associated with a 30.0% increase in the odds of presenting low cognitive engagement.

Regarding model fit, the Hosmer–Lemeshow tests were non-significant for all models, indicating no evidence of poor calibration. The corresponding *p*-values were 0.681, 0.898, and 0.726 for behavioral, emotional, and cognitive engagement in the urban models, and 0.277, 0.514, and 0.283, respectively, in the rural models. Nagelkerke’s R^2^ values were 0.059, 0.080, and 0.073 for the urban models and 0.177, 0.205, and 0.249 for the rural models, respectively.

## 4. Discussion

The present study examined the association between weekly MVPA and school engagement among adolescents from rural and urban schools in southern Spain. In subgroup-specific analyses, active adolescents from urban schools had significantly higher cognitive engagement than their inactive peers, with an 11.4% higher adjusted mean score and a small-to-moderate effect size. Consistently, lower weekly MVPA was associated with higher odds of low cognitive engagement in urban adolescents, with each one-day decrease in weekly MVPA increasing the odds of low cognitive engagement by 30.0%. However, these subgroup-specific findings should be interpreted cautiously, as the formal interaction analyses showed no significant differences between rural and urban contexts for any engagement dimension. Taken together, these findings indicate that the observed significance in the urban subgroup cannot be interpreted as evidence of a stronger or context-specific association, and the secondary hypothesis that school context would moderate the relationship between MVPA and school engagement was therefore not supported.

The specific association between MVPA and cognitive engagement is consistent with previous evidence suggesting that physical activity may contribute to educationally relevant cognitive and motivational outcomes. It is coherent with recent findings from southern Spain showing that less favorable sedentary behavior patterns were associated with lower cognitive engagement, whereas more structured sedentary behaviors were positively related to behavioral and cognitive engagement [7]. In addition, previous systematic review and meta-analytic evidence suggest a small positive association between physical activity and school engagement in young people, although findings appear to be heterogeneous [25]. Similarly, physical activity and fitness have been positively linked to cognitive function and academic achievement in children and adolescents [13,37], while the potential effects of physical activity on cognitive and mental health have been explained through neurobiological, psychosocial, and behavioral mechanisms [15]. More recent evidence suggests that cognitively engaging physical activity interventions may improve executive functions in children and adolescents, particularly inhibitory control and working memory, which are closely related to students’ ability to sustain effort, resist distractions, and use learning strategies during academic tasks [18,19]. At the psychological level, regular MVPA may also support self-regulation and self-efficacy, two factors directly linked to persistence and perceived competence in learning situations [22,23]. Therefore, MVPA may be more closely related to cognitive engagement, which involves students’ mental investment, effort, persistence, and strategic involvement in learning, than to behavioral or emotional engagement [18,22].

The absence of significant associations for behavioral and emotional engagement may reflect the multidimensional nature of school engagement, whose dimensions are related but not interchangeable [21,26,29]. In this regard, recent evidence suggests that behavioral, emotional, and cognitive engagement may show different patterns of association with health-related behaviors and may also be shaped by different individual and contextual factors, including teacher–student relationships, school environment, and students’ personal strengths [7,28]. In this sense, behavioral engagement may be more dependent on observable participation, classroom structure, school rules, and teacher expectations, whereas emotional engagement may be more closely related to belonging, affective connection with school, perceived support, and interpersonal relationships. This interpretation is supported by longitudinal evidence showing that family, teacher, and peer support have distinct predictive effects on affective, cognitive, and behavioral engagement, as well as by recent findings indicating that engagement dimensions differ in their degree of subject and time specificity [27,32]. Moreover, recent meta-analytic evidence suggests that different types of engagement may serve different educational functions, with emotional engagement being more strongly linked to motivation and well-being, and behavioral engagement more closely related to achievement-related outcomes [30]. From this broader perspective, associations between MVPA and behavioral or emotional engagement may be more indirect and contingent on interpersonal and instructional conditions, while the different engagement dimensions appear to serve partly distinct educational functions [30,32]. Accordingly, the non-significant findings for behavioral and emotional engagement may reflect the dimensional specificity of school engagement rather than a contradiction of previous evidence.

Although significant associations were observed only in the urban subgroup, the non-significant interaction analyses indicate that this pattern cannot be interpreted as evidence of a differential association between rural and urban adolescents. Nevertheless, contextual characteristics of rural and urban environments may theoretically influence how MVPA relates to educational functioning [24]. In urban settings, where adolescents’ movement behaviors, screen-time patterns, and access to green or open spaces may differ from those of rural adolescents, sport and structured physical activity may represent one of the main opportunities to disconnect from academic tasks and screen-based behaviors, making MVPA a more salient resource for cognitive engagement [8,9,33]. In contrast, rural adolescents’ school engagement may be shaped by different everyday routines and social relationships, including informal social interaction, community connectedness, and perceived support in their surrounding environment, which could make cognitive engagement less dependent on MVPA alone [8,10,45]. From this perspective, physical activity in rural contexts may operate more as part of a broader social and relational environment than as an isolated predictor of cognitive engagement, particularly given the relevance of family and friend support for MVPA levels among rural adolescents [11]. However, these contextual explanations remain hypothetical because the relevant environmental and social variables were not directly assessed in the present study.

Accordingly, the absence of significant associations among rural adolescents should not be interpreted as evidence that MVPA is unrelated to school engagement in rural settings. Rather, the relationship may be more strongly embedded within the broader social and environmental context in which physical activity occurs. Previous research in rural adolescents has highlighted the importance of family and peer support for physical activity participation [11], while family, teacher, and peer support have also been identified as relevant predictors of different dimensions of school engagement [32]. This suggests that social relationships may influence both behaviors simultaneously, potentially making the independent contribution of MVPA to engagement less evident when these contextual influences are not considered. In addition, characteristics of the physical and social environment, such as neighborhood resources, access to amenities, and community support, appear to be particularly relevant for physical activity among rural youth [8,34]. Therefore, the educational implications of MVPA in rural settings may depend not only on how much activity adolescents perform, but also on the social support, opportunities, and contexts accompanying that activity. Future research should therefore include larger rural samples and use longitudinal or multilevel designs that directly assess these contextual factors to determine whether they mediate or modify the association between MVPA and school engagement.

From an educational perspective, the present findings suggest that the association between MVPA and cognitive engagement may have practical relevance, although its magnitude should be interpreted cautiously. A small-to-moderate difference in cognitive engagement may reflect potentially relevant group-level differences in students’ psychological investment in learning, persistence, effort, and strategic involvement, all of which are relevant to educational functioning. This interpretation is supported by previous meta-analytic evidence linking school engagement with academic achievement and subjective well-being [29]. Nevertheless, the observed difference cannot be directly translated into improvements in grades, attendance, or other objective educational outcomes, and no established threshold is available to determine what constitutes an educationally meaningful change in cognitive engagement. Therefore, regular opportunities for MVPA may represent a potentially supportive component of educational practice, but the practical significance of this association should be confirmed through longitudinal and intervention studies before specific educational recommendations are established.

### Limitations and Strengths

Several limitations should be acknowledged. First, the cross-sectional design precludes establishing the temporal direction or causality of the associations between MVPA and school engagement. Therefore, reverse or bidirectional relationships cannot be ruled out, as adolescents with higher school engagement may also be more likely to participate regularly in MVPA. Second, schools were recruited through convenience sampling based on their availability and willingness to participate. Consequently, the sample may not be fully representative, and the generalizability of the findings is limited. Moreover, schools that agreed to participate may have differed systematically from those that did not, introducing potential selection bias. In addition, the inclusion of adolescents exclusively from southern Spain restricts the transferability of the results to other geographical regions, educational systems, and age groups. Third, MVPA and school engagement were assessed using self-reported instruments, which may be affected by recall bias or social desirability. Fourth, the dichotomization of the continuous school engagement scores using the sample median may have resulted in a loss of statistical information and does not represent an established educational threshold. Additionally, the smaller rural subsample may have reduced the statistical power of the stratified analyses. Because participants were nested within only six schools, multilevel modeling or cluster-robust standard errors were not applied, as estimates may be unstable with so few clusters. Consequently, potential within-school dependence cannot be excluded. Finally, the absence of contextual variables, including access to sport facilities, active transport opportunities, socioeconomic status, and school-level factors, may have introduced residual confounding, as these conditions could influence both opportunities for MVPA and school engagement. Despite these limitations, this study has several strengths. It included adolescents from both rural and urban schools, allowing the association between MVPA and school engagement to be examined according to school context. It also used a validated multidimensional measure of school engagement, which enabled behavioral, emotional, and cognitive engagement to be analyzed separately rather than as a global construct. Furthermore, the analyses were adjusted for relevant covariates, including age, sex, and BMI, and MVPA was examined using both categorical and continuous approaches, through ANCOVA to compare active and inactive adolescents and binary logistic regression to estimate the risk of low engagement associated with lower weekly MVPA.

## 5. Conclusions

In conclusion, subgroup-specific analyses showed that weekly MVPA was associated with cognitive engagement among adolescents attending urban schools. Active urban adolescents showed higher cognitive engagement than their inactive peers, and lower weekly MVPA was associated with a greater likelihood of low cognitive engagement in this group. These findings highlight the potential relevance of physical activity for cognitive aspects of school engagement, such as effort, persistence, and psychological investment in learning. No significant associations were observed for behavioral or emotional engagement, suggesting that the relationship between physical activity and school engagement may vary across engagement dimensions. Importantly, the interaction analyses did not identify significant rural–urban differences. Therefore, the findings observed in the urban subgroup should not be interpreted as evidence of a context-specific effect or as evidence that school context modifies the association between MVPA and school engagement. Given the cross-sectional design, these findings should be interpreted as associative rather than causal. From an educational perspective, providing regular opportunities for MVPA may be relevant during adolescence; however, this implication is based on cross-sectional evidence and requires confirmation through longitudinal and experimental research before causal or practice-oriented conclusions can be drawn.

## Figures and Tables

**Table 1:** Descriptive characteristics of the study sample according to school context.

Variables	All (*n* = 282)		Urban (*n* = 199)		Rural (*n* = 83)		
	Mean	SD	Mean	SD	Mean	SD	*p*
Sex	—	—	—	—	—	—	0.992
Girls	50.2%	—	50.2%	—	50.1%	—	—
Boys	49.8%	—	49.8%	—	49.9%	—	—
Age (years)	13.29	1.63	13.22	1.71	13.47	1.42	0.243
Weight (kg)	53.49	13.17	52.81	12.93	55.13	13.67	0.178
Height (m)	1.60	0.10	1.59	0.11	1.60	0.08	0.331
BMI (kg/m^2^)	20.84	3.90	20.67	3.78	21.24	4.18	0.261
BMI-for-age category (%)	—	—	—	—	—	—	0.483
Thinness	2.1%	—	1.5%	—	3.6%	—	—
Normal weight	64.1%	—	66.2%	—	59.0%	—	—
Overweight	24.2%	—	23.7%	—	25.3%	—	—
Obesity	9.6%	—	8.6%	—	12.0%	—	—
Weekly MVPA (days/week)	4.08	1.73	4.05	1.77	4.14	1.66	0.705
Active (≥5 days/week)	36.9%	—	37.7%	—	34.9%	—	0.764
Inactive (<5 days/week)	63.1%	—	62.3%	—	65.1%	—	—
School Engagement	—	—	—	—	—	—	—
Behavioral Engagement	4.25	0.65	4.27	0.62	4.22	0.70	0.546
Emotional Engagement	3.56	0.90	3.58	0.89	3.52	0.94	0.581
Cognitive Engagement	3.12	0.76	3.14	0.76	3.09	0.77	0.675

Note. BMI = body mass index; MVPA = moderate-to-vigorous physical activity. Values are presented as mean and standard deviation unless otherwise indicated. Differences between urban and rural adolescents were examined using Student’s *t*-tests for continuous variables and chi-square tests for categorical variables.

**Table 2:** Binary logistic regression models for the association between weekly MVPA and school engagement according to school context.

				Rural			Urban		
		*n* (%)	*p*	OR	95% CI	*n* (%)	*p*	OR	95% CI
Behavioral engagement	High	53 (63.9%)		1	Referent	135 (68.2%)		1	Referent
	Low	30 (36.1%)	0.494	1.122	0.806–1.565	63 (31.8%)	0.163	1.135	0.951–1.355
Emotional engagement	High	63 (76.8%)		1	Referent	155 (79.1%)		1	Referent
	Low	19 (23.2%)	0.256	1.245	0.853–1.818	41 (20.9%)	0.075	1.211	0.981–1.493
Cognitive engagement	High	61 (75.3%)		1	Referent	146 (74.5%)		1	Referent
	Low	20 (24.7%)	0.148	1.355	0.898–2.041	50 (25.5%)	0.009	1.300	1.067–1.585

Note. MVPA = moderate-to-vigorous physical activity; OR = odds ratio; CI = confidence interval. Low school engagement was defined as scoring below the median in each engagement dimension. ORs indicate the increased odds of low engagement for each one-day decrease in weekly MVPA. Models were adjusted for age, BMI, and sex. Due to missing data, the sample size varied slightly across models.

## Data Availability

The datasets underpinning this study’s results are not accessible to the public due to ethical and confidentiality considerations. As this research forms part of a broader project involving various collaborators, safeguarding participant anonymity was essential to secure their participation. In alignment with ethical standards and to preserve the privacy of respondents, data sharing is not permitted.

## Abbreviations

The following abbreviations are used in this manuscript:

ANCOVA	Analysis of covariance
BMI	Body mass index
CFI	Comparative fit index
CI	Confidence interval
MVPA	Moderate-to-vigorous physical activity
OR	Odds ratio
RMSEA	Root mean square error of approximation
SD	Standard deviation
SEM	School engagement measure
SRMR	Standardized root mean square residual
